# Mitigation of salt stress on low temperature in bermudagrass: resistance and forage quality

**DOI:** 10.3389/fpls.2022.1042855

**Published:** 2022-10-28

**Authors:** Xiuwen Zhou, Yanling Yin, Guangyang Wang, Erick Amombo, Xiaoning Li, Ying Xue, Jinmin Fu

**Affiliations:** Coastal Salinity Tolerant Grass Engineering and Technology Research Center, Ludong University, Yantai, China

**Keywords:** bermudagrass, low temperature, salt, photosynthesis, antioxidant, glyoxalase

## Abstract

Climate change causes plants encountering several abiotic stresses simultaneously. Responses of plants to a single stress has been comprehensively studied, but it is hard to speculated infer the effects of stress combination based on these researches. Here, the response mechanism of bermudagrass to low temperature and salt treatment was investigated in this study. The results showed that low temperature (LT) treatment decreased the relative growth rate, chlorophyll fluorescence transient curve, biomass, and crude fat content of bermudagrass, whereas low temperature + salt (LT+S) treatment greatly undermined these declines. Furthermore, at 6 h and 17 d, the expression levels of *glyoxalase I* (*GLYI*), *Cu-Zn/superoxide dismutase* (*Cu-Zn/SOD*), *peroxidase 2* (*POD2*), and *oxidative enzyme 1*(*CAT1*) in roots were considerably higher in the low temperature + salt treatment than in the low temperature treatment. Low temperature stress is more detrimental to bermudagrass, but mild salt addition can mitigate the damage by enhancing photosynthesis and improving the expression of antioxidant system genes (*Cu-Zn/SOD*, *POD2* and *CAT1*) and glyoxalase system *GLYI* gene in roots. This study summarized the probable interaction mechanism of low temperature and salt stress on bermudagrass, which can provide beneficial reference for the growth of fodder in cold regions.

## 1 Introduction

Bermudagrass, as a popular forage and warm season turfgrass, shows limited growth and begins to wilt when the daily mean temperature goes below 12°C, and then it enters the dormancy period when the temperature reaches 7-10°C (Munshaw et al., 2004; Huang et al., 2019). Low temperature restricts the establishment of bermudagrass in cold region. In addition, bermudagrass has advantages in the restoration of saline-alkali land due to resistance against salt stress (Xie et al., 2017). Therefore, bermudagrass often suffers from combination both of low temperature and salt stress.

Low temperature and salt stress lead to shared or specific physiological, metabolic and genes expression responses. It is well known that low temperature and salt stress restrain plant growth and reduce photosynthesis (Allen and Ort, 2001; Hasdai et al., 2006; Li et al., 2019; Zulfiqar and Ashraf, 2021b; Zulfiqar et al., 2022b). Plants experienced chloroplast structural damage, chlorophyll degradation, stomatal closure and enzyme activity decrease during both low temperature and salt stress (Meloni et al., 2003; Nowicka et al., 2018). Oxidative stress resulted from over accumulation of reactive oxygen species (ROS) and osmotic stress appear in plant response to both low temperature and salt stress (Hasanuzzaman et al., 2020a; Zulfiqar and Ashraf, 2021a; Zulfiqar and Ashraf, 2022a). Both oxidative and osmotic stress have negative effects on plant metabolism, molecular biosynthesis and cell viability (Mahajan and Tuteja, 2005; Liang et al., 2018; Zulfiqar et al., 2019; Munns et al., 2020; Zhao et al., 2021). Furthermore, both low temperature and salt stress cause alteration in ion homeostasis, which destroy the biological activity of membrane and some enzymes (Fürtauer et al., 2019; Shao et al., 2020). Differently, salt stress results in a toxic concentration of Na^+^ and inhibits absorption of K^+^ (Kim et al., 2007; Yang and Guo, 2018). Many specific ions transported genes are responsible for transportation of Na^+^ and K^+^, such as *SOS* (salt overly sensitive) family genes and *HKT* (high affinity transporter) (Li et al., 2019). Low temperature disturbs transmembrane H^+^ gradient by changing the activity of a H^+^ pumping protein, H^+^-ATPase (Ponce-Pineda et al., 2021).

Plants are equipped with fine tune resistant mechanisms to counter low temperature and salt stress (Banerjee et al., 2018). Osmoprotectants such as proline and soluble sugar are biosynthesized to remit molecular denaturation (Fürtauer et al., 2019; Zulfiqar et al., 2019; Munns et al., 2020). Plants have a complex antioxidant system, which includes enzymatic system as well as the glutathione-ascorbic acid (GSH-ASA) cycle (Bela et al., 2015). Antioxidant systems are activated to scavenge ROS. For example, antioxidant enzymes such as SOD (superoxide dismutase), POD (enzymes peroxidase) and CAT (oxidative enzyme) and their encoding genes are upregulated in the early stress response (Hasanuzzaman et al., 2020a; Hasanuzzaman et al., 2020b; Shao et al., 2020; Zulfiqar et al., 2020a; Zulfiqar and Ashraf, 2021a; Jabeen et al., 2022; Zulfiqar and Ashraf, 2022a). SOD has the ability to catalyze the conversion of superoxide anions to H_2_O_2_ and O_2_, which is the primary substance for scavenging free radicals in living things. H_2_O_2_ is scavenged by enzymes CAT and POD through synergistic action to maintain a stable level of free radicals in plants (Yan et al., 2010). In addition, glyoxalase (GLY) system, closely related to GSH metabolism, was reported to participate in stress responses in the past, such as salt and heavy metal stress (Yadav et al., 2005; Singla-Pareek et al., 2006; Roy et al., 2008; Singla-Pareek et al., 2008). GLY is a type of intracellular enzyme found in both prokaryotes and eukaryotes which is mostly sublocalized in the cytoplasm and organelles. GLY system has long been known in animals and is assumed to be engaged in a variety of tasks, including cell division, proliferation, and protection against oxoaldehyde toxicity (Thornalley, 1990). In plants, salt stress increased GlLYI activity, which is accompanied by MG detoxification and decrease in GSH concentration(Parvin et al., 2019).

At the moment, the global climate and environment are rapidly changing, and plants are being subjected to an increasing number of abiotic stresses (Zulfiqar and Hancock, 2020b; Zulfiqar et al., 2021c). However, plants are endowed with unique physiological responses under combined stress which are completely different from those under single stress (Bai et al., 2018; Henriet et al., 2019). Many studies have reported that the negative effects of stress interactions on crop productivity are much higher than when different stress components are applied alone (Mittler and Blumwald, 2010; Suzuki et al., 2014). The relative water content of tomato (Rong et al., 2017) and the biomass of Phragmites Karka (Abideen et al., 2022) were significantly decreased under compound stress. Drought and heat stress can lead to a significant reduction in Arabidopsis growth, but their combined stress has a more deleterious effect (Vile et al., 2012). However, other studies have reported the beneficial effects of the interaction of two different stresses applied simultaneously (Suzuki et al., 2014). For example, drought stress may offer protection against O_3_ damage in plants (Biswas and Jiang, 2011; Zhang et al., 2019) and resulted in reduced susceptibility to powdery mildew and Botrytis cinerea (Achuo et al., 2006). Combined stress of drought and salt can reduce boron toxicity in plants (Liu et al., 2018)

Previous research has typically focused on either low temperature or salt stress, with only a few studies have been done related to the combined effect of low temperature and salt stress on plants. Bermudagrass is a valuable forage grass and warm season turfgrass with a high salt tolerance. It is critical to investigate the physiological and molecular mechanisms of bermudagrass under the combined stress of low temperature and salt, in order to improve tolerance and lengthen the growth cycle. In this study, we used physiological and molecular methods to assess the effects of low temperature and salt stress on growth traits, photosynthesis, forage quality, and stress-related gene expression in this study. As a result, the goals of this study were to: (1) investigate the interaction of low temperature and salt on bermudagrass; and (2) elucidate the synergistic mechanism of low temperature and salt.

## 2 Materials and methods

### 2.1 Plant materials and growth condition

In this investigation, bermudagrass (*Cynodon dactylon*(L.) Pers.) “A12359” was used. On 6 July 2019, the bermudagrasses with uniformly cut roots were transplanted from the fields into plastic containers (4 cm in diameter and 21 cm deep) which filled with commercially available plant media, 36 pots in total were transplanted. The plants were kept in a controlled greenhouse with natural light (240 µmolm^-2^s^-1^), a 30/24°C average day/night temperature, and 50% relative humidity. The grasses were fertilized with 1/2 Hoagland nutrient solution (NH_4_H_2_PO_4_ (0.5 mM), KNO_3_ (2.5 mM), Ca (NO_3_)_2_.4H_2_O (2.5 mM), MgSO_4_.7H_2_O (1 mM), H_3_BO_3_ (1.43 mg), ZnSO_4_.7H_2_O (0.11 mg), CuSO_4_·5H_2_O (0.04 mg), MnCl_2_.4H_2_O (0.91 mg), H_2_MoO_4_ (0.05 mg), Fe-EDTA (0.04 mM)) twice a week. After three weeks, 24 potted plants were selected and clipped consistently to 14 cm, then placed to a plant incubator on 27 July 2019.

### 2.2 Treatments and experimental design

Control (CK), salt (S), low temperature (LT), low temperature +salt (LT+S) were the four experimental treatments. There were six pots for each treatment and a random block design was used to minimize the impact of environmental conditions. The LT and LT+S treatments were 15/10°C (day/night), while the others were 35/30°C (day/night). S and LT+S treatments were grown for 3 d with 1.0 percent salt solution, then 1.5 percent for 3 d, and finally 2.0 percent salt solution, until the material exhibited a substantial phenotype difference (17 d). Dilution of sea water yielded the saline solution. In the plant incubator, the plants were kept at 240µmolm^-2^s^-1^ photosynthetically active radiation, a 16-hour photoperiod, and 50% relative humidity. At 17 d following treatment (August 13, 2019), leaves and roots were harvested for physiological investigation, and gene expression levels in the leaves and roots were measured at 6 h and 17 d. These plants’ chlorophyll fluorescence and chlorophyll content were also measured at 3, 6 and 17 d of age.

### 2.3 Methods

#### 2.3.1 Phenotypic determination

Root length: All treated plants was measured with a ruler after roots had been washed and drained on absorbent paper.

Plant height: On d 3 (July 30, 2019), 6 (August 2, 2019) and 17 (August 13, 2019), the plant height was measured with a ruler.

Plant samples were heated in an oven at 105°C for 30 minutes before being dried to constant weight at 80°C and weighed on a 1/10000 precision balance.

#### 2.3.2 Chlorophyll content

We took 0.1 g of fresh leaves and cut them short (about 0.5 cm long), then placed them in a 15 ml centrifuge tube with 10 ml dimethyl sulfoxide and shaded them for 2-3 d. In a centrifuge tube, 1ml chlorophyll extract and 2 ml dimethyl sulfoxide were mixed and poured into a colorimetric dish. The absorbance is measured at 663 nm and 645 nm wavelengths using dimethyl sulfoxide as the blank. Three potted plants were collected per treatment.

#### 2.3.3 Chlorophyll a fluorescence transient and the JIP-Test

A pulse-amplitude modulation fluorometer was used to create a fluorescence transient of chlorophyll a. (PAM2500, Heinz Walz GmbH). After 30 minutes of dark adaptation, the shoots (same position of plants in each treatment) were exposed to a red light of 3,000μmol photons m^-2^s^-1^. There were three replicates from different potted plants of each treatment. JIP-test was used as **Table 1** to better analyze the OJIP curve.

**Table 1:** Photosynthetic parameters deduced by the JIP-test analysis of fluorescence transients.

	1.0% Salt				1.5% Salt				2.0% Salt				Definitions
	CK	S	LT	LT+S	CK	S	LT	LT+S	CK	S	LT	LT+S	
*DATA EXTRACTED FROM THE RECORDED OJIP FLUORESCENCE TRANSIENT CURVES*													
F_0_=F20_μs_	0.54b	0.55b	0.65a	0.63ab	0.53ab	0.47b	0.61a	0.58a	0.64a	0.57a	0.56a	0.58a	Fluorescence at time t after onset of actinic illumination
F_K_	1.09a	1.12a	1.08a	0.99a	1.18a	1.00b	0.87b	0.88a	1.48a	1.26b	0.68d	0.81c	Fluorescence value at 300μs
F_J_	1.32ab	1.39a	1.23ab	1.13b	1.38a	1.27a	0.97b	0.99b	1.60a	1.42b	0.87c	0.71d	Fluorescence value at the J-step of OJIP
F_I_	1.78a	1.78a	1.59b	1.38c	1.75a	1.69a	1.16b	1.11b	1.85a	1.68b	0.74d	0.96c	Fluorescence value at the I-step of OJIP
F_P_=F_M_	1.91a	1.89a	1.68b	1.47c	1.87a	1.81a	1.18b	1.25b	1.94a	1.76b	0.79c	1.03d	Fluorescence value at the peak of OJIP test
M_0_	1.61a	1.72a	1.66a	1.71a	1.93a	1.57b	1.82b	1.82ab	0.93a	0.93a	0.79b	0.83b	Approximate value of the initial slope of fluorescence transient curves
V_J_	0.57a	0.63a	0.56a	0.60a	0.64a	0.60a	0.64a	0.61a	0.74a	0.71a	0.67ab	0.62b	Relative variable fluorescence at J-step
Area	24.57a	22.58a	21.56a	18.83a	21.4b	24.79a	14.82c	15.98b	18.64a	18.31a	10.53c	14.7b	the area above the chlorophyll fluorescence curve between Fo and Fm
N	49.90b	46.06b	61.70a	61.27a	48.26b	48.43b	75.47a	71.02a	49.95b	50.01a	150.60a	112.94ab	number of Q_A_ redox turnovers until Fm is reached
*SPECIFIC ENERGY FLUXES (PER ACTIVE PSII REACTION CENTER)*													
ABS/RC	3.91b	3.86b	4.81a	5.05a	4.25b	3.55c	5.99a	5.55a	5.21b	4.82b	11.35a	7.82ab	Absorbed photon flux per RC
TR_0_/RC	2.82a	2.75a	2.95a	2.89a	3.04a	2.63b	2.87ab	2.99ab	3.51a	3.23a	3.36a	3.33a	Trapped excitation flux (leading to QA reduction) per RC
ET_0_/RC	1.20a	1.03a	1.30a	1.17a	1.11a	1.06a	1.05a	1.16a	0.93a	0.95a	1.12a	1.16a	Electron transport flux (further than QA-) per RC
*QUANTUM YIELDS AND EFFICIENCIES/PROBABILITIES*													
φP_0_= TR_0_/ABS	0.72a	0.71a	0.62b	0.57b	0.71a	0.74a	0.48b	0.54b	0.68a	0.67a	0.30c	0.43b	Maximum quantum yield for primary photochemistry
ΨE_0_= ET_0_/TR_0_	0.43a	0.37a	0.44a	0.41a	0.36a	0.40a	0.36a	0.39a	0.17b	0.30b	0.33ab	0.38a	Efficiency/probability with which a PSII trapped electron is transferred from Q_A_ to Q_B_
φE_0_= ET_0_/ABS	0.31a	0.27a	0.27a	0.24a	0.26a	0.30a	0.18b	0.21b	0.18a	0.20a	0.10b	0.16a	Quantum yield of the electron transport flux from Q_A_ to Q_B_
σR_0_= RE_0_/ET_0_	0.23b	0.22b	0.20b	0.27a	0.25ab	0.23b	0.35a	0.35a	0.27b	0.26b	0.64a	0.46ab	Efficiency/probability with which an electron from Q_B_ is transferred until PSI acceptors
φR_0_= RE_0_/ABS	0.65a	0.57a	0.55a	0.65a	0.67a	0.70a	0.63a	0.77a	0.05b	0.05b	0.06ab	0.08a	Quantum yield for reduction of end electron acceptors at the PSI acceptor side
γRC	0.21a	0.21a	0.17b	0.17b	0.19b	0.22a	0.14c	0.15c	0.16a	0.18a	0.08b	0.12b	Probability that a PSII Chl molecule functions as RC
RC/ABS	0.26a	0.26a	0.21b	0.21b	0.24b	0.28a	0.17c	0.18c	0.19a	0.21a	0.13b	0.09b	Number of QA reducing RCs per PSII antenna Chl
*PERFORMANCE INDEXES (PI, COMBINATION OF PARAMETERS)*													
PI_ABS_	0.40a	0.32ab	0.22ab	0.15b	0.29b	0.43a	0.08c	0.12c	0.12a	0.15a	0.02b	0.06b	PI (potential) for energy conservation from exciton to the reduction of intersystem electron
PI_total_	0.12a	0.09a	0.06a	0.06a	0.09b	0.13a	0.04d	0.06c	0.05a	0.05a	0.04a	0.05a	PI (potential) for energy conservation from exciton to the reduction of PSI end acceptors

Each parameter is calculated according to pervious method (Yusuf et al., 2010). Subscript ‘‘0’’ denotes that the parameter refers to the onset of illumination. Values are given as the average of 3 replicates, and different letters denote statistic significant difference at P < 0.05 among the treatments by Tukey’s multiple range tests.

#### 2.3.4 Measurement of nutritive value

Na^+^ and K^+^: We weighed 0.1 g of the material into the desiccating tube, added 10 ml H2SO4, and placed it in the graphite digesting device. The amount of Na^+^ and K^+^ was determined using a flame spectrophotometer after the desiccated sample was diluted 100 times. For each treatment, three biological replicates collected from different potted plants.

#### 2.3.5 Quantitative RT -PCR analysis

For each treatment, three biological replicates were taken from different potted plants. Trizol reagent (Invitrogen, America) was used to isolated total RNA from about 0.1 g samples of leaves and roots. DNasel was used to remove contaminating genomic DNA from RNA. A UV spectrophotometry NanoDrop was used to examine the RNA concentration and purity (Thermo Fisher Scientific, Lenexa, Ks, USA). Using a Hifair III 1st Strand cDNA Synthesis SuperMix for qPCR with genome-DNA-removing enzyme, 2.5 ug RNA was reverse transcribed to cDNA (Yesen, Nanjing, China). The qPCR was carried out on a Quant Studio 6 detection system (ABI, Forster City, CA, USA) with a SYBR green PCR mix (Takara, RR420A, Shika, Japan). The following was the real-time PCR program: 95°C for 5 minutes; 40 cycles of 95°C for 10 seconds and 60°C for 30 seconds. **Table 2** contains a list of primers. For gene expression level analysis, the bermudagrass Actin gene was used as an inner control, and the comparative Ct method was used.

**Table 2:** Primer sequences for RT-PCR amplification analysis in bermudagrass.

Gene name	Forward primers	Reverse primers
GLY1	ATGTGTGTGACCGACTTTGCC	GAAGTCGTGCGTCTCGAAGGA
Cu-Zn/SOD	TAGCCCAGTTGTCGTGAGCCT	GGATTTGACCGTTGACTGGCG
POD2	TCGCTTCTGCTGGACGATGAC	GGGGGTCGAAGAAGTTGGGTA
CAT1	TCTCAGACAAGGACCGACTCA	GAGAAGCGGACGAATACTGGT
ACTIN	TCTGAAGGGTAAGTAGAGTAG	ACTCAGCACATTCCAGCAGAT

### 2.4 Data analysis

All treatments were repeated three times. All the data was subjected to analysis of variance (AVOVA) with the Duncan’s multiple range tests means at a significant level of *P*<0.05 using the statistical package SPSS 16.0, Origin Pro 9.0 and Excel 2019 for Windows.

## 3 Results

### 3.1 Effects of low temperature and salt stress on the growth and physiological characteristics of bermudagrass

**Figure 1:**
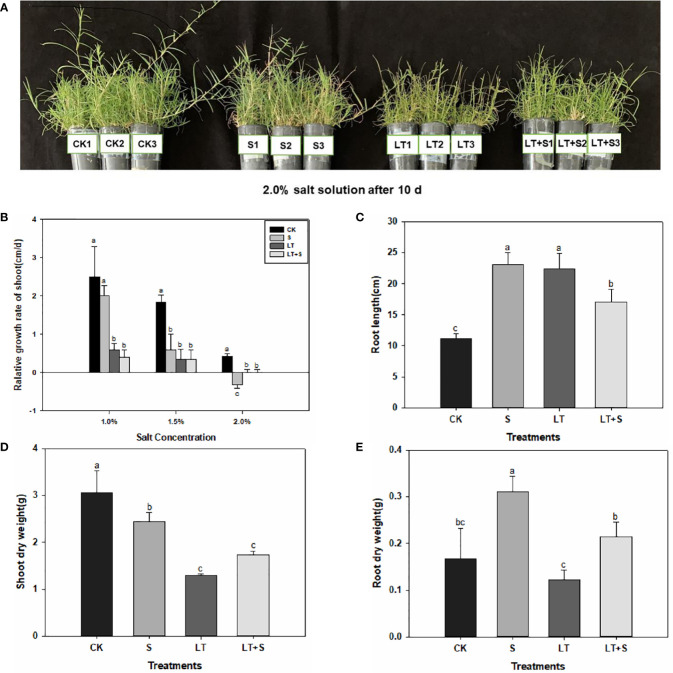
Bermudagrass phenotypic features under low temperature and salt stress. **(A)** Image of bermudagrass plants. **(B)** Relative growth rate of shoot. **(C)** Root length. **(D)** Shoot dry weight. **(E)** Root dry weight. Bermudagrass was transplanted into black plastic tubes after being clipped to a uniform length at the root. The plant height was reduced to the same height after a time of cultivation in a greenhouse. Duncan’ s multiple range tests show that different letters above the same columns imply statistically significant differences at P < 0.05.

Bermudagrass growth was hindered under the three stress regimes. However, the development of bermudagrass under the LT+S treatment was superior to that under the LT treatment (**Figure 1A**). LT treatment and LT+S treatment drastically decreased the relative growth rate of shoot compared to control (**Figure 1B**). In comparison to the relative growth rate of shoots under 1.0 percent salt concentration, the relative growth rate of shoots under 1.5 percent and 2.0 percent salt concentration was further reduced with the continuous increase of salt concentration. However, there was no statistically significant difference in relative growth rate between combined stress and LT treatment (**Figure 1B**). When plants were subjected to salt and low temperature stress, the length of their roots exhibited an obvious elongation character (**Figure 1C**). Plant shoots dry weight decreased in all three stress treatments when compared to the control, and there was no difference between the LT and LT+S treatments (**Figure 1D**). The root dry weight increased significantly after salt treatment alone, and combined stress alleviates single low temperature damage (**Figure 1E**).

### 3.2 Effects of low temperature and salt stress on the photosynthetic efficiency of bermudagrass

**Figure 2:**
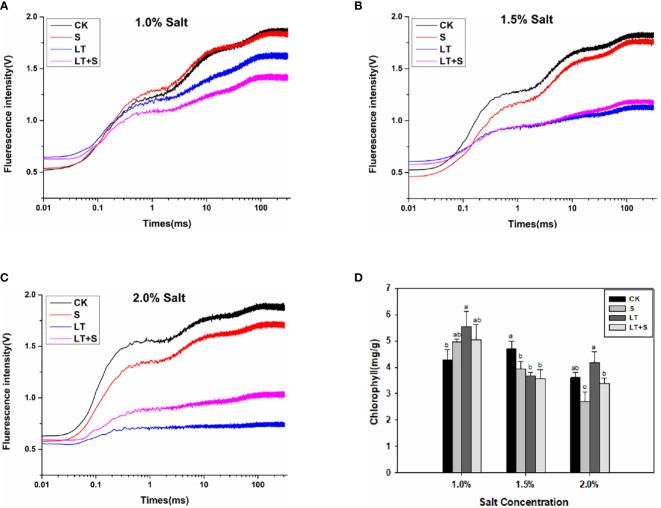
Effects of salt and cold treatment on photosynthetic parameters. **(A–C)**. Transient changes in chlorophyll fluorescence in bermudagrass leaves under 1.0 percent **(A)**, 1.5 percent **(B)** and 2.0 percent **(C)** salt treatment. **(D)** Chlorophyll content. Different letters above the same columns represent statistically significant differences at P < 0.05 (Duncan’ s multiple range test).

The OJIP fluorescence transient curves were plotted in **Figure 2** to show the effects of low temperature and salt on the photosynthetic efficiency of bermudagrass. The chlorophyll fluorescence transient response curve did not change significantly when treated with 1.0 percent salt compared to the control, but it was significantly reduced when treated with low temperature (**Figure 2A**). Furthermore, in the 2.0 percent range, the LT+S treatment curve rose with increasing salt concentration and was significantly higher than the LT treatment alone (**Figure 2C**). The values of basic fluorescence parameters were extracted from the recorded OJIP curve, and several structural and functional parameters were calculated and analyzed (**Table 1**). When salt content was less than 2.0 percent, area, φP_0_ and φE_0_ was greatly reduced under low temperature compared to the control, but significantly increased under LT+S stress compared to the LT treatment. All of these findings suggest that salt treatment can help plants recover from the effects of low temperature treatment. At salt concentrations of 1.0 percent and 1.5 percent, there was no difference in chlorophyll content between the LT+S treatment and the LT treatment, but at 2.0 percent, the chlorophyll content of the LT+S treatment was much lower than that of the LT treatment (**Figure 2D**). To summarize, combined stress affects photosynthetic performance by regulating PSII rather than chlorophyll content.

### 3.3 Effects on ionic homeostasis of bermudagrass under low temperature and salt stress

**Figure 3:**
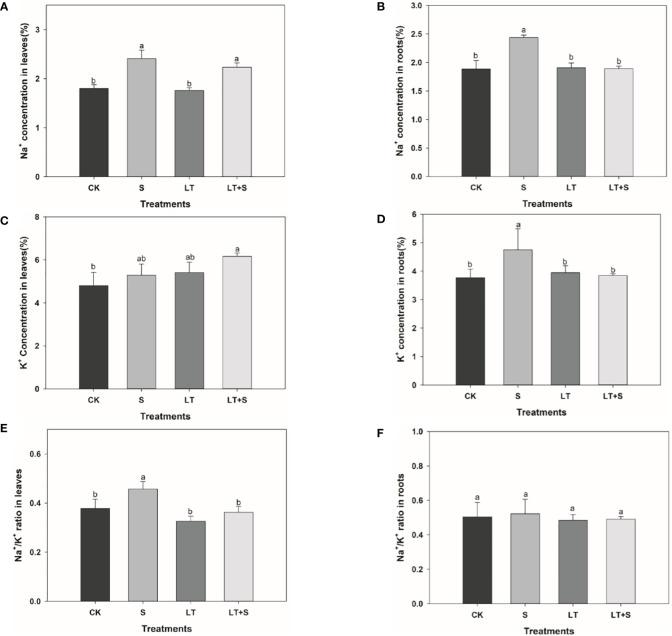
The bar chart shows the average Na+ concentration **(A, B)**, K+ concentration **(C, D)**, and Na+/K+ ratio **(E, F)** of bermudagrass under each treatment, with three replicates per treatment. Different letters above the same columns indicate statistic significant difference at P < 0.05 (Duncan’ s multiple range test).

We measured the concentration of Na^+^ and K^+^ to further investigate the effects of salt and low temperature on bermudagrass. In leaves, the Na^+^ concentration increased significantly under salt stress compared with the control, and there was no difference between LT+S and S treatments (**Figure 3A**). However, K^+^ concentration in leaves increased remarkably under LT+S treatment, while there was no difference under low temperature and salt stress alone compared with the control (**Figure 3C**). In roots, Na^+^ and K^+^ concentration increased significantly under salt stress compared with the control, while them decreased significantly under LT+S treatment compared with the salt treatment (**Figures 3B**, **3D**). Finally, compared with salt stress, the ratio of Na^+^/K^+^ in leaves was significantly decreased under combined stress and there was no difference in Na^+^/K^+^ ratio in roots under all stress conditions (**Figures 3E, F**).

### 3.4 Effects on resistance related genes under low temperature and salt stress

**Figure 4:**
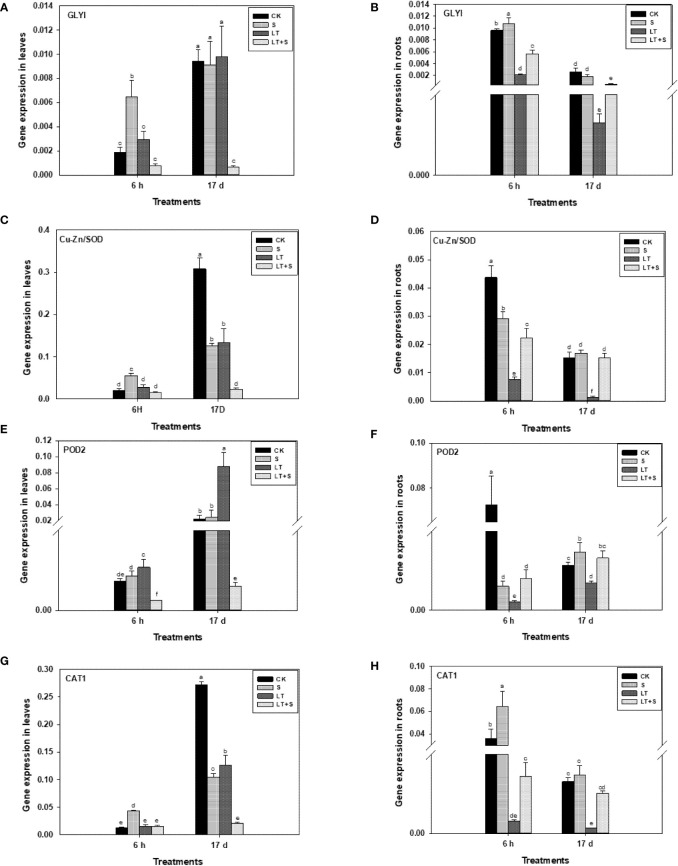
Glyoxalase and antioxidant enzyme related genes transcriptional level in bermudagrass under low temperature and salt stress. **(A, C, E, G)** represents genes transcriptional level in leaves and Figure **(B, D, F–H)** represents genes transcriptional level in roots. Different letters above the same columns indicate statistic significant difference at P < 0.05 (Duncan’ s multiple range test).

*GLYI*, *Cu-Zn/SOD*, *POD2* and *CAT1* expressions were measured to investigate the role of GLY and antioxidant system under low temperature and salt stress. *GLYI* was upregulated in both leaves and roots after 6 h of salt treatment, but it was no longer up-regulated after 17 d of salt treatment (**Figures 4A, B**). At 6 h or 17 d, LT treatment had no effect on *GLYI* in leaves but significantly reduced *GLYI* in roots (**Figures 4A, B**). *GLYI* expression in roots was significantly increased after 6 h and 17 d of LT+S treatment when compared to LT treatment (**Figure 4B**). Similarly, antioxidant-related genes (*Cu-Zn/SOD*, *POD2*, and *CAT1*) were downregulated in roots after 6 h and 17 d of LT treatment (**Figures 4D, F, H**). The combination of salt and LT, on the other hand, suppressed the expression of antioxidant-related genes. *Cu-Zn/SOD* and *CAT1* expression levels in leaves exhibited no difference between control and low temperature treatment after 6 h, but were down-regulated after 17 d (**Figures 4C**, **G**). In comparison to the control treatment, the expression of *POD2* in leaves rose considerably after 17 d of low temperature treatment (**Figure 4E**). When compared to low temperature treatment alone, combined stress does not further the expression of these genes. In conclusion, combined stress reduces low temperature stress in roots *via* regulating these genes.

### 3.5 Effects on forage nutritive value of bermudagrass under low temperature and salt stress

**Figure 5:**
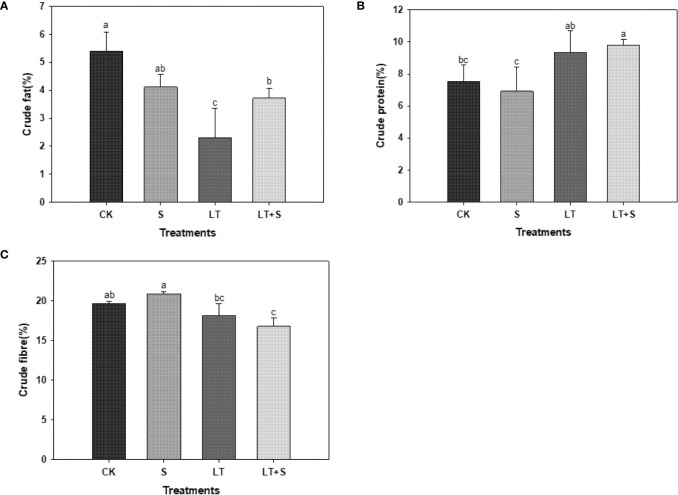
The bar chart shows the average of crude fat **(A)**, crude protein **(B)**, and crude fiber **(C)** content of bermudagrass under each treatment, with three replicates per treatment. Different letters above the same columns indicate statistic significant difference at P < 0.05 (Ducan’ s multiple range test).

We measured the quality indexes to further investigate the effects of salt and low temperature on bermudagrass. The effect of salt treatment alone on the nutritive value of forage was insignificant. The crude fat content was significantly lower in the LT and LT+S treatments compared to the control, but significantly higher in the LT+S treatment compared to the LT treatment alone, which was consistent with the fluorescence curve results (**Figure 5A**). Unlike crude fat, crude protein was significantly increased under LT+S conditions (**Figure 5B**). Only the LT+S treatment significantly reduced crude fiber (**Figure 5C**). It’s worth mentioning that under the combined stress condition, the forage quality of the bermudagrass did not degrade any more compared to the low temperature treatment, but the crude fat rose.

## 4 Discussion

This article looked into the defensive mechanism of bermudagrass under low temperatures and salt stress. Plants response to stress is a complex process including morphology, physiology, and biochemistry (Huang et al., 2019). Low temperature-treated bermudagrass showed reduced relative growth rate in shoots, shoot dry weight, and increased root length in previous research (Esmaili and Salehi, 2012), which matched our findings (**Figures 1B, E**). From the standpoint of plant growth phenotype, bermudagrass under the LT+S treatment is superior to that of low temperature treatment, the root dry weight reflects the same situation. Relative growth rate, shoot dry weight and root length under low temperature and low temperature + salt treatment shows no discernible differences (**Figures 1A-E**). All of this suggests that the combined stress did not produce more substantial harm to the plants; rather, a moderate amount of salt may have mitigated the damage caused by low temperatures. Maintaining proper balance of Na^+^/K^+^ and higher K^+^ concentration is considered as an important mechanism for plants to response to salt stress (Evelin et al., 2009). Lower Na^+^/K^+^ is a marker of salinity tolerance for plants (van Zelm et al., 2020). In salt-tolerant plants, K^+^ efflux can be significantly inhibited to maintain stable Na^+^/K^+^ and reduce salt stress injury (Yang and Guo, 2018). In our study, Na^+^/K^+^ remained stable and K^+^ concentration increased in root under salt stress (**Figures 3D**, **F**), so it can be inferred that bermudagrass A12359 has a certain salinity tolerance, which is the reason that it can alleviate the damage of low temperature to bermudagrass at the high salt concentration (2.0%).

Low temperature stress affects a variety of physiological processes in plants, the most susceptible of which is photosynthesis (Stitt and Hurry, 2002). We looked at photosynthetic indexes at the third, sixth and seventeenth d to learn more about how bermudagrass protects itself under low temperatures and different salt concentrations. The PSII reaction center becomes sensitive under stress, and the OJIP fluorescence transient curves and chlorophyll fluorescence characteristics can accurately reflect PSII’s physiological status (Chen et al., 2021). The electron transport activities (PSI and PSII) of the chloroplast thylakoid membrane were found to be dramatically reduced at low temperatures, with PSII being more vulnerable to cold pressure than PSI (Shi et al., 2016). The chlorophyll fluorescence transient response curve decreased under the three stress conditions as processing time at low temperature and salt concentration increased, compared to control, in our studies, but it decreased more noticeably under the LT treatment, and the fluorescence curve is higher under the LT+S treatment than the LT treatments when the salt concentration reaches 1.5 percent and 2.0 percent (**Figures 2B, C**).The findings revealed that an optimal salt concentration could help to relieve photosynthetic physiology in cold-stressed plants (**Figures 2A, C**). Low temperature stress causes a drop in E0, which is mostly influenced by alterations in the PSII receptor side (Van Heerden et al., 2003). φE_0_ represents the reaction center of absorbed light quantum yield for electron transfer, and the higher the value, the more stressed the plants are. In this study, φE_0_ significantly decreased under LT stress while improving under LT+S treatment, and the same changes were observed in area and φP_0_. All of these results suggest that when LT+S was used instead of LT stress, electron transfer efficiency improved, and the effect of low temperature stress alone on plant photosynthesis was reduced.

Plants respond to salt, severe temperatures, and other factors through the GLY system, which consists of GlyI, GlyII, and GSH (coenzyme). The detoxification of methylglyoxal, a by-product of carbohydrate metabolism, is its primary physiological role (Roy et al., 2008). In this study, since the expression levels of GLYI and antioxidant oxidases related genes change early under stress conditions (Wang et al., 2020; Zhang et al., 2021), the gene expression levels were measured once after 6 h of treatment, and then again at the end of the experiment (17d), so as to observe the gene expression changes. Under salt, mannitol and heavy metal stress, *GLYI* expression in mustard was dramatically increased (Veena et al., 1999). *GLYI* was considerably elevated in both roots and leaves after 6 h of salt treatment in our experiment (**Figures 4A, B**), which is consistent with earlier research. Low temperature causes mechanical constraints-membrane damage, whereas salinity causes malignancy by disrupting the ion and osmotic balance of cells (Mahajan and Tuteja, 2005). Osmotic stress can particularly promote the production of GLYI, at which point the GLY system is triggered to repair the damage (Inoue et al., 1998). Low temperature stress was found to increase the levels of reactive oxygen species (ROS) and lower enzyme antioxidant activity in plants (Aroca et al., 2005; Sasaki et al., 2007), affecting the antioxidant system. In our study, antioxidant enzyme genes (*Cu-Zn/SOD*, *POD2*, and *CAT1*) in roots were significantly down-regulated under low temperature stress (**Figures 4D**, **F**, **H**). When exposed to low temperature stress alone, the expression of *GLYI* in leaves does not rise and even decreases dramatically in roots, and several indicators reveal that the injury to plants is highest at this time. However, when compared to low temperature treatment, the expression level of *GLYI* was higher during LT+S stress, indicating that the GLY system was activated again, reducing the damage caused by low temperature. Furthermore, the GLY system raises the level of free reduced glutathione, which is necessary for the removal of harmful reactive oxygen species (e.g., H_2_O_2_) and organic peroxides that accumulate in stressed plants, as well as the maintenance of other antioxidants (Frendo et al., 2013). Despite the roles of antioxidant enzymes and GLY in stress are clearly, we indicated that these genes may involve in stress acclimation to several different environmental adversity.

Crude protein and crude fiber are two important indexes for measuring the nutritional value of herbage, as well as important contents for improving herbage quality. The first selection factors for high quality forage are high crude protein content and low crude fiber content (Barth, 2012). Previous research has shown that plants in low temperature environments reduce structural carbohydrate content while increasing soluble carbohydrate and protein content to minimize plant harm (Vo and Johnson, 2001). In our experiment, compared to the control, bermudagrass protein content increased significantly under LT and LT+S treatment (**Figure 5B**). This phenomenon could be explained by the fact that the LT treatment was set at 10 -15°C and the cells were subjected to low temperature stress but did not reach the point where the fluidity of the cell membrane becomes weak and rigidity increases. At this point, the cell membrane’s phase transition reduces the ability of the membrane protein to bind to phospholipids and causes it to become free protein, resulting in an increase in protein content in the plant. Crude fat is a nutrient with a high calorific value that can enhance feed palatability (Li et al., 2019). According to the test results, the crude fat content was lowest under low temperature treatment and significantly higher under low temperature + salt treatment (**Figure 5A**), proving once again that appropriate salt can mitigate the damage caused by low temperature.

## 5 Conclusion

At present, plants are subjected to more and more abiotic stresses (Zulfiqar and Hancock, 2020b; Zulfiqar et al., 2021c), and the physiological responses under combined stress and single stress are completely different. Therefore, the changes of phenotype, photosynthesis, Na^+^, K^+^, gene expression and forage quality under single salt, low temperature stress and combined stress were analyzed in this study to explore the interaction between low temperature and salinity on bermudagrass. It was found that low temperatures cause more damage to bermudagrass, but moderate salt addition can mitigate the damage by enhancing photosynthesis, improving the expression of antioxidant system genes (*Cu-Zn/SOD*, *POD2* and *CAT1*) and glyoxalase system *GLYI* gene in roots and thus improves forage quality. This provides putative pathways improving turfgrass and forage tolerance to combination stress.

## Data availability statement

The original contributions presented in the study are included in the article/supplementary material. Further inquiries can be directed to the corresponding author.

## Author contributions

All authors contributed largely to the work presented in this article. Conceived and designed the experiments: JF. Performed the experiments XZ, GW, XL, YX. Analyzed the data: XZ, YY. Language modification: EA. Wrote the paper: XZ, YY. All authors contributed to the article and approved the submitted version.

## Funding

This work was supported by National Key R&D Program of China (2019YFD0900702) and Agricultural Variety Improvement Project of Shandong Province (2019LZGC010).

## Conflict of interest

The authors declare that the research was conducted in the absence of any commercial or financial relationships that could be construed as a potential conflict of interest.

## Publisher’s note

All claims expressed in this article are solely those of the authors and do not necessarily represent those of their affiliated organizations, or those of the publisher, the editors and the reviewers. Any product that may be evaluated in this article, or claim that may be made by its manufacturer, is not guaranteed or endorsed by the publisher.

## Abbreviations

CK, control treatment; S, salt treatment; LT, low temperature treatment; LT+S, low temperature + salt treatment; GLY, glyoxalase; SOD, superoxide dismutase; POD, peroxidase; CAT, oxidative enzyme; MG, methylglyoxal; GSH, glutathione; ASA, ascorbic acid; SOS, salt overly sensitive; HKT, high affinity transporter.
